# Potential for the lung recruitment and the risk of lung overdistension during 21 days of mechanical ventilation in patients with  COVID-19 after noninvasive ventilation failure: the COVID-VENT observational trial

**DOI:** 10.1186/s12871-022-01600-0

**Published:** 2022-03-04

**Authors:** Andrey I. Yaroshetskiy, Sergey N. Avdeev, Mikhail E. Politov, Pavel V. Nogtev, Victoria G. Beresneva, Yury D. Sorokin, Vasily D. Konanykhin, Anna P. Krasnoshchekova, Zamira M. Merzhoeva, Natalia A. Tsareva, Natalia V. Trushenko, Irina A. Mandel, Andrey G. Yavorovskiy

**Affiliations:** 1 Sechenov First Moscow State Medical University (Sechenov University), 8/2, Trubetskaya str., 119991 Moscow, Russia; 2 Pirogov Russian National Research Medical University, 1, Ostrovitianova str, 117997 Moscow, Russia

**Keywords:** COVID-19, PEEP, Volutrauma, Lung recruitability, Lung strain, Acute respiratory distress syndrome

## Abstract

**Background:**

Data on the lung respiratory mechanics and gas exchange in the time course of COVID-19-associated respiratory failure is limited. This study aimed to explore respiratory mechanics and gas exchange, the lung recruitability and risk of overdistension during the time course of mechanical ventilation.

**Methods:**

This was a prospective observational study in critically ill mechanically ventilated patients (*n* = 116) with COVID-19 admitted into Intensive Care Units of Sechenov University. The primary endpoints were: «optimum» positive end-expiratory pressure (PEEP) level balanced between the lowest driving pressure and the highest SpO_2_ and number of patients with recruitable lung on Days 1 and 7 of mechanical ventilation. We measured driving pressure at different levels of PEEP (14, 12, 10 and 8 cmH_2_O) with preset tidal volume, and with the increase of tidal volume by 100 ml and 200 ml at preset PEEP level, and calculated static respiratory system compliance (C_RS_), PaO_2_/FiO_2_, alveolar dead space and ventilatory ratio on Days 1, 3, 5, 7, 10, 14 and 21.

**Results:**

The «optimum» PEEP levels on Day 1 were 11.0 (10.0–12.8) cmH_2_O and 10.0 (9.0–12.0) cmH_2_O on Day 7. Positive response to recruitment was observed on Day 1 in 27.6% and on Day 7 in 9.2% of patients. PEEP increase from 10 to 14 cmH_2_O and VT increase by 100 and 200 ml led to a significant decrease in C_RS_ from Day 1 to Day 14 (*p* < 0.05). Ventilatory ratio was 2.2 (1.7–2,7) in non-survivors and in 1.9 (1.6–2.6) survivors on Day 1 and decreased on Day 7 in survivors only (*p* < 0.01). PaO_2_/FiO_2_ was 105.5 (76.2–141.7) mmHg in non-survivors on Day 1 and 136.6 (106.7–160.8) in survivors (*p* = 0.002). In survivors, PaO_2_/FiO_2_ rose on Day 3 (*p* = 0.008) and then between Days 7 and 10 (*p* = 0.046).

**Conclusion:**

Lung recruitability was low in COVID-19 and decreased during the course of the disease, but lung overdistension occurred at «intermediate» PEEP and VT levels. In survivors gas exchange improvements after Day 7 mismatched C_RS_.

**Trial registration:**

ClinicalTrials.gov, NCT04445961. Registered 24 June 2020—Retrospectively registered.

**Supplementary Information:**

The online version contains supplementary material available at 10.1186/s12871-022-01600-0.

## Background

Most patients with COVID-19-associated acute respiratory failure fulfil the criteria of the acute respiratory distress syndrome (ARDS) and often require invasive mechanical ventilation [1–18]. In these patients it may be crucial to understand the principal features of gas exchange abnormalities, respiratory mechanics and lung recruitability in order to provide an appropriate adjustment of the positive end-expiratory pressure (PEEP), the tidal volume and the use of recruitment maneuvers. Gattinoni L et al. recently proposed two phenotypes of COVID-19-related ARDS: L-phenotype (low lung elastance and low recruitability) and H-phenotype (high lung elastance and high recruitability) at the late stage [1]. On the contrary, in comparative studies the COVID-related ARDS was similar to the primary non-COVID-related ARDS [2, 3, 18]. However, observational studies have shown high variability in the optimum PEEP levels and in the lung recruitability in these patients, and covered predominantly the first 7 days of mechanical ventilation [3–5, 7, 9–11, 15–17].

The goal of the study was to investigate respiratory mechanics and gas exchange during the first 21 days of mechanical ventilation with the aim for selection of the «optimum» positive end-expiratory pressure (PEEP), evaluation of recruitability and a risk of volutrauma in COVID-19-associated acute respiratory failure.

## Methods

### Study design

This was a prospective observational clinical study (ClinicalTrials.gov NCT04445961) conducted in intensive care units (ICUs) at three hospitals of Sechenov University (Moscow, Russia) from May 1 to August 14, 2020. The study was approved by the Institutional Ethics Committee (reference number: 16–20). Written informed consent was waived owing to the observational nature of the study.

### Patients

All mechanically ventilated patients (both invasive and non-invasive) were daily screened for eligibility. We included patients with COVID-19-associated respiratory failure requiring invasive mechanical ventilation after noninvasive ventilation (NIV) failure. Exclusion criteria were: 1)peripheral oxygen saturation (SpO_2_) > 93%, no visible work of auxiliary respiratory muscles (sternocleidomastoid and scalene), no fatigue on conventional oxygen therapy (oxygen flow < 15 l/min) or non-invasive ventilation; 2) life-threatening heart rhythm abnormalities and/or systolic blood pressure < 80 mmHg despite norepinephrine at a dose > 2 µg/kg/min; 3) primary lung diseases (e.g. interstitial lung diseases, lung emphysema) or tumor metastases in lungs; 4) chronic decompensated diseases with extrapulmonary organ dysfunction (tumor progression, liver cirrhosis, congestive heart failure); 6) atonic coma. We withdrew patients from the analysis if ICU stay was less than 24 h for any reason.

### Measurements

At the start of the study all patients were on mechanical ventilation in the assisted pressure-controlled volume-guaranteed mode in supine position with the tidal volume (VT) set at 6–8 ml/kg of the predicted body weight (PBW) and positive end-expiratory pressure set at 8 cmH_2_O, inspiratory time 0,8–1,1 s to prevent air trapping at exhalation, respiratory rate (RR) set at 16–28 to reach arterial carbon dioxide tension (PaCO_2_) 35–50 mmHg and inspiratory fraction of oxygen (FiO_2_) set at minimal level to reach SpO_2_ 93–96%. Patients were sedated with a propofol infusion up to the Richmond Agitation-Sedation Score (RASS) -3–4 points and paralyzed if they had inspiratory swings on pressure–time curve and/or visible work of auxiliary respiratory muscles besides RASS-4.

We measured plateau pressure (Pplat) with the inspiratory hold maneuver for 3 s at PEEP levels of 14, 12, 10 and 8 cmH_2_O and calculated driving pressure (DP) as Pplat-PEEP and static respiratory system compliance (C_RS_) on Days 1, 3, 5, 7, 10, 14, 21 and 28 (if applicable) («PEEP trial»). We set the PEEP level at mentioned time points at the balance point of the lowest DP and the highest SpO_2_. We observed patients after the PEEP setting for at least 15 min to determine the highest SpO_2_ and optimal FiO_2_. After the PEEP setting, we increased tidal volume by 100 ml and 200 ml in 2 steps, respectively, and measured Pplat on each step with DP and C_RS_ calculation (on Days 1 and 7 as part of recruitment maneuver)(«volume trial»). For the correct calculation of C_RS_ during a volume increase we computed the «normalized» C_RS_ by dividing the tidal volume in ml/kg of the predicted body weight to driving pressure.

Also, on Days 1 and 7, we used the recruitment maneuver (RM) doubling the tidal volume for 15 respiratory cycles at a preset PEEP level (the doubled tidal volume was reached by several steps of 100 ml each). Before the maneuver we set FiO_2_ that corresponds to SpO_2_ 90%. Prior to and at the end of the maneuver we measured the plethysmography variability index (PVI) by Radical-7 monitor (Masimo Corp, Irvine, CA, USA). We defined RM as effective if SpO_2_ rose to 95% and higher in 5 min after RM.

Patients were placed in the prone position for at least 16 h per day if it led to an increase in SpO_2_ by more than 5% except for patients with body mass index > 40 kg/m^2^ (they were placed in lateral positions) and patients in whom the prone position led to an increase in driving pressure. The ventilation mode was switched to the Pressure Support mode if a patient was conscious or sedated up to RASS 0–2, had a stable respiratory and hemodynamic state, no visible work of auxiliary respiratory muscles and a stable respiratory pattern after switching. The pressure support level was set according to Pplat and corrected to achieve the Tobin index (respiratory rate/VT) of less than 70. Tracheostomy was performed on the 3rd day of mechanical ventilation.

### Laboratory tests

Before the PEEP and volume trials we measured partial pressure of oxygen in arterial blood (PaO_2_), partial pressure of carbon dioxide in arterial blood (PaCO_2_), arterial pH, and end-expiratory carbon dioxide tension (PetCO_2_), and calculated PaO_2_/FiO_2_ ratio, alveolar dead space (VDalv/VT) according to Bohr-Enghoff equation and ventilatory ratio (VR) [19].

Routine blood examinations included 1) complete blood count, 2) coagulation profile—fibrinogen, activated partial thromboplastin time, international normalized ratio and D-dimers, 3) serum biochemical tests (C-reactive protein, albumin, creatinine, blood urea nitrogen, total bilirubin, alanine transaminase and aspartate transaminase, lactate dehydrogenase, electrolytes and serum ferritin). The frequency of tests was determined by the attending physician (everyday, as usual).

### Endpoints and statistical analysis

The primary endpoints were: 1. «Optimum» PEEP level on Days 1 and 7 of mechanical ventilation balanced between the lowest DP and the highest SpO_2_; 2. Number of patients with recruitable lung defined as SpO_2_ changed from 90 to 95% and more after recruitment maneuver on Days 1 and 7 of mechanical ventilation.

Secondary endpoints included: 1. «Optimum» PEEP level on Days 3, 5, 10, 14 and 21 (set as described above); 2. Driving pressure at different PEEP levels (8, 10, 12, 14 mbar) and different tidal volumes (initial, + 100 ml and + 200 ml) at a set PEEP level on Days 1, 3, 5, 7, 10, 14, 21 of the mechanical ventilation; 3. Alveolar dead space on Days 1, 3, 5, 7, 10, 14, 21 of the mechanical ventilation; 4. Plethysmography variation index before PEEP and volume trials and at the end of the recruitment maneuver on Days 1 and 7 of the mechanical ventilation or during the volume trial on Days 3, 5,10 and 21; 5. PaO_2_/FiO_2_ ratio and ventilatory ratio on Days 1, 3, 5, 7, 10, 14, 21 of the mechanical ventilation.

Descriptive statistics included proportions for categorical and median (interquartile range) for continuous variables. No imputation was made for missing data. To assess differences between survivors and non-survivors, we performed the Mann–Whitney U test for continuous variables, and Chi-square or Fisher exact test for categorical variables. The Friedman test was used for variable dynamics within group. A two-sided *p* < 0.05 was considered statistically significant. Statistical analyses were performed using SPSS Statistics version 19.0 (IBM, Armonk, NY, USA).

## Results

**Fig. 1:**
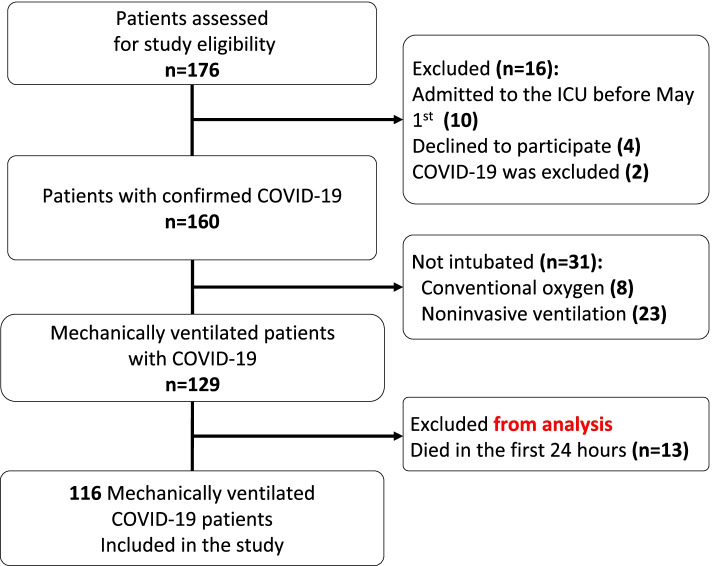
The study cohort selection

We consecutively identified 176 and enrolled 116 patients (Fig. 1). Baseline demographic and laboratory characteristics, comorbidities and medications of all patients and subgroups of survivors and non-survivors are summarised in Table 1.

**Table 1:** Patient's demographic characteristics, comorbidities, medications and laboratory values at inclusion

	**Overall** (***n*** = 116)	**Survivors** (***n*** = 17)	**Non-Survivors** (***n*** = 99)	***p***
**Demographics**				
Age, years	70.0 [60.3–78.0]	66.0 [59.0–81.5]	70.0 [61.0–78.0]	0.645
Males, n (%)	64 (55.2)	7 (41.2)	57.0 (57.6)	0.209
Height, cm	175.0 [165.5–180.0]	167.0 [165.0–179.0]	175.0 [167.0–180.0]	0.186
BMI, kg/m^2^	31.2 [28.4–34.3]	32.5 [30.7–34.5]	31.0 [28.2–34.3]	0.078
Comorbidities, n (%)				0.751
Hypertension	87 (75.0)	12 (70.6)	75 (75.8)	
Diabetes Mellitus	45 (38.8)	6 (35.3)	39 (39.4)	
Ischemic heart disease	36 (31.0)	5 (29.4)	31 (31.3)	
Congestive heart failure	9 (7.8)	0 (0)	9 (9.1)	
Atrial fibrillation	19 (16.4)	0 (0)	19 (19.2)	
Obesity	40 (34.5)	6 (35.3)	34 (34.3)	
COPD/Asthma	11 (9.5)	3 (17.6)	8 (8.1)	
History of stroke	7 (6.0)	0 (0)	7 (70.7)	
Cerebrovascular disease	8 (6.9)	1 (5.9)	7 (70.7)	
History of Cancer	9 (7.8)	1 (5.9)	8 (8.1)	
History of MI	12 (10.3)	0 (0)	12 (12.1)	
Pulmonary hypertension	3 (2.6)	0 (0)	3 (3.0)	
Smoking history:				0.645
Former smokers, n (%)	9 (7.8)	1 (5.9)	8 (8.1)	
Active smokers, n (%)	3 (2.6)	0 (0)	3 (3.0)	
ACE inhibitors or ARB, n (%)	82 (70.7)	11 (64.7)	71 (71.7)	
Time from onset, days	13 [9-18]	12 [9-14]	13 [9-21]	0.103
SOFA score	6 [5-8]	6 [5-7]	6 [5-9]	0.345
NIV duration, days	3.0 [2.0–5.8]	3.0 [1.5–5.5]	3.0 [2.0–6.0]	0.831
**Lung CT**				
Lung involvement, %	83 [78–87]	79 [75–83]	83 [80–87]	0.003
Lung consolidation, %	12 [9,10-15]	10 [8-13]	12 [9-16]	0.133
**Treatment, n(%)**				
Hydroxychloroquine	110 (94.8)	17 (100.0)	93 (93.9)	
Lopinavir/ritonavir	23 (19.8)	3 (17.6)	15 (15.2)	
Dexamethasone (8–20 mg/day)	116 (100.0)	17 (100.0)	99 (100.0)	1.000
UFH or LWH «low dose»	5 (4.3)	1 (5.9)	4 (4.0)	
UFH or LWH «high dose»	111 (95.7)	16 (94.1)	95 (95.9)	
Anticytokine therapy				
Tocilizumab	18 (15.5)	5 (29.4)	13.0 (13.1)	0.258
Sarilumab	5 (4.3)	1 (5.9)	4.0 (4.0)	
Tofacitinib	1 (0.9)	1 (5.9)	0 (0)	
**Laboratory values**				
WBC, 10^9^/l	11.1 [8.1–15.3]	9.6 [7.8–15.8]	11.5 [8.4–15.2]	0.494
Lymphocytes, 10^9^/l	0.7 [0.4–0.9]	0.5 [0.5–0.8]	0.7 [0.4–0.9]	0.431
D-dimer, mcg/ml	3.3 [1.6–6.8]	3.3 [2.0–6.0]	3.3 [1.6–7.3]	0.751
Fibrinogen, g/l	7.5 [5.7–9.4]	6.9 [5.3–10.6]	7.6 [5.7–9.4]	0.785
Creatinine, mcg/l	97.0 [75.8–136.7]	89.0 [66.0–114.2]	99.0 [76.0–139.0]	0.240
LDH, U/l	996.5 [833.3–1347.8]	859.5 [716.3–1137.8]	1025.5 [842.5–1428.5]	0.114
CRP, mg/l	160.1 [100.3–246.9]	163.1 [118.3–234.1]	157.2 [98.0–257.8]	0.984

Data presented as medians [interquartile range] or n (%) where appropriate. Differences between groups: Mann–Whitney U-test, Chi-square or Fisher exact test where appropriate. *p*-value: comparison between survivors and non-survivors

Lung involvement is defined as the proportion of the lung infiltrates including ground-glass opacities, crazy paving, and consolidation on high-resolution CT scan to whole lung volume. Lung consolidation is defined as the proportion of the lung consolidation volume to lung infiltrates volume. We used medications included in «Prophylaxis, Diagnostics, and Treatment of patients with COVID-19. Temporary Clinical Guideline» issued by the Russian Ministry of Health for that time (versions 1–3).

*Abbreviations*: *BMI *body mass index; *COPD* chronic obstructive lung disease; *MI* myocardial infarction; *ACE* angiotensin-converting enzyme; *ARB* angiotensin-receptor blocker; *SOFA* sequential organ failure assessment; *NIV* noninvasive ventilation; *CT* computed tomography; *UFH* unfractionated heparin; *LWH* low weight heparin; *WBC* white blood cells; *LDH* lactate dehydrogenase; *CRP* C-reactive protein

**Fig. 2:**
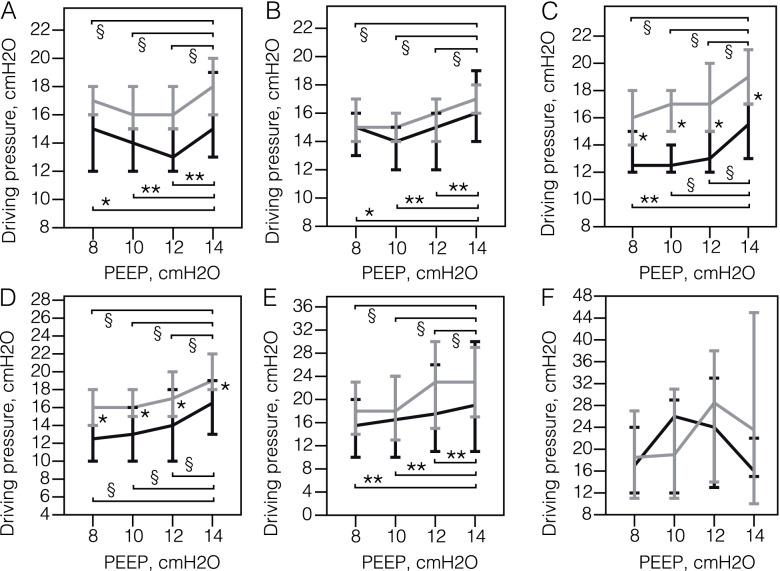
The driving pressure at different positive end-expiratory pressure levels (PEEP) («PEEP trial») in survivors and non-survivors during 21 days of the mechanical ventilation. **A** Day 1. **B** Day 3. **C** Day 5. **D** Day 7. **E** Day 14. **F** Day 21. Data on survivors (black) and non-survivors (grey) is presented as medians and 95% confidence in-tervals. The x-axis represents positive end-expiratory pressure levels in cmH2O . * *p*-value < 0.05, comparison within subgroup of survivors and non-survivors (Friedman test) ; ** *p*-value < 0.01, comparison within subgroup of survivors and non-survivors (Friedman test) ;§ *p*-value < 0.001, comparison within subgroup of survivors and non-survivors (Friedman test)

The «PEEP trial» (Fig. 2, Table E1) showed that the PEEP increase from 10 to 14 cmH_2_O resulted in a significant increase in driving pressure on Days 3, 5, 7, 10 and 14 both in survivors and non-survivors. Driving pressure decreased with PEEP increase from 8 to 12 cmH_2_O in survivors on Day 1. Driving pressure levels were significantly higher in non-survivors on Days 5, 7 and 10. Driving pressure rose at equal PEEP levels during the study period and it reached 48 cmH_2_O at PEEP 14 cmH_2_O in 1 non-survivor at day 28.

**Fig. 3:**
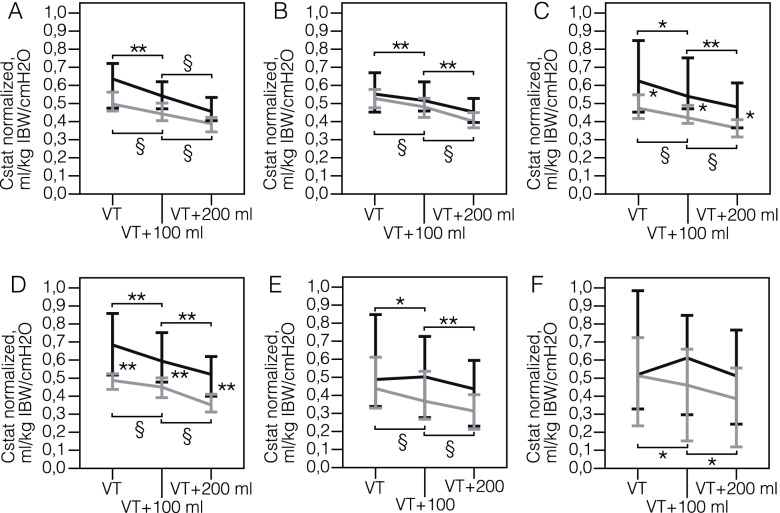
Normalized static compliance at preset tidal volume and during tidal volume increase («volume trial») in survivors and non-survivors during 21 days of the mechanical ventilation. **A** Day 1. **B** Day 3. **C** Day 5. **D** Day 7. **E** Day 14. **F** Day 21. Data on survivors (black) and non-survivors (grey) is presented as medians and 95% confidence in-tervals. The x-axis represents three points: initial tidal volume, tidal volume increased by 100 ml and tidal volume increased by 200 ml. Normalized static compliance calculated dividing the tidal volume in ml/kg of the ideal body weight to driving pressure.* *p*-value < 0.05, comparison within subgroup of survivors and non-survivors (Friedman test);** *p*-value < 0.01, comparison within subgroup of survivors and non-survivors (Friedman test);§ *p*-value < 0.001, comparison within subgroup of survivors and non-survivors (Friedman test)

Volume increase by 100 ml and 200 ml from the set volume and PEEP («volume trial») resulted in a significant decrease in normalized Cstat on Days 1, 3, 5, 7, 10 and 14 in all patients and on Day 21 in non-survivors (Fig. 3, Table E1). Differences between normalized Cstat in survivors and non-survivors were significant on Days 5, 7 and 10. At day 21 minimal normalized Cstat in non-survivor at tidal volume + 200 ml was 8 cmH_2_O/ml and driving pressure reached 74 cmH_2_O (Table E1). Lung computed tomography (CT) data before intubation revealed significant differences in the volume of lung involvement between survivors and non-survivors that were reflected by differences in respiratory mechanics seen in Figs. 2 and 3. We found that modified Clinical Pulmonary Infection Score (CPIS) was significantly higher in non-survivors on days 3, 5, and 7, but the score reached the cut-off value of 6 points only after Day 10. On Day 10 only 3 of 17 survivors and 15 of 32 non-survivors had modified CPIS >  = 6 points. Only 3 patients in the non-survivors group reached CPIS 7 points on Day 10. We think that low compliance in these patients may be at least partially explained by ventilator-associated pneumonia.

**Fig. 4:**
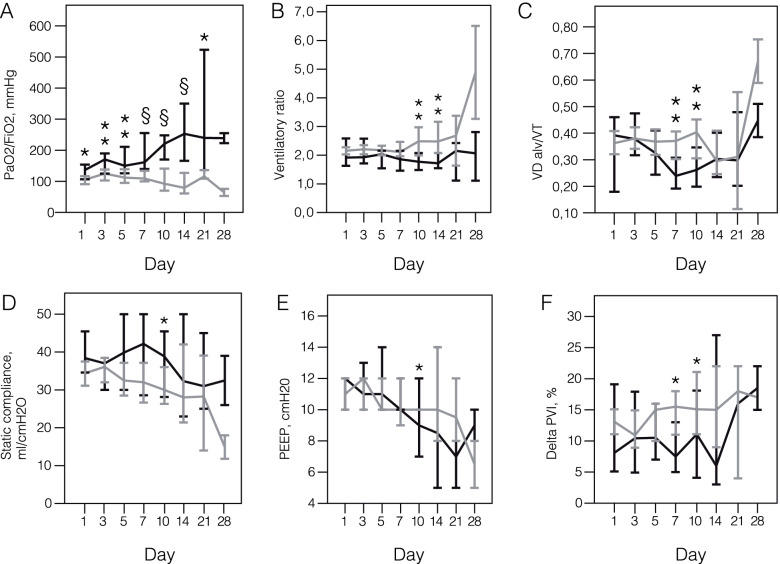
The gas exchange, respiratory mechanics and plethysmogram varia-bility during 28 days of the mechanical ventilation in survivors and non-survivors. **A** PaO2/FiO2. **B** Ventilatory ratio. **C** Alveolar dead space to tidal volume ratio. **D** Static compliance of the respiratory system. **E** The «optimum» positive end ex-piratory pressure balanced between the lowest driving pressure and the highest pe-ripheral oxygen saturation (SpO2). **F** Change in plethysmogram variability index during the recruitment maneuver (Days 1 and 7) or the tidal volume increase by 200 ml (on Days 3, 5,10,14, 21 and 28). Data on survivors (black) and non-survivors (grey) is presented as medians and 95% confidence in-tervals. The x-axis represents days after initiation of the mechanical ventilation. Abbreviations: PaO2- partial pressure of oxygen in arterial blood; FiO2—inspiratory oxygen fraction; VDalv—alveolar dead space: VT- tidal volume; PEEP—positive end-expiratory pressure; PVI—ple-thysmogram variability index; SpO2 peripheral oxygen saturation.* *p*-value < 0.05, comparison between survivors and non-survivors (Mann-Whitney U test) ;** *p*-value < 0.01, comparison between survivors and non-survivors (Mann-Whitney U test) ;§ *p*-value < 0.001, comparison between survivors and non-survivors (Mann-Whitney U test)

Static compliance at a preset tidal volume and PEEP showed a mild-to-moderate decrease and was not significantly different within and between survivors and non-survivors in dynamics (Fig. 4, Table 2). Driving pressure differed significantly between survivors and non-survivors on Days 5, 7 and 10 and didn’t change significantly within survivors but increased in non-survivors on Day 21 (*p* = 0.046).

**Table 2:** Respiratory parameters, ventilatory settings, and adjunctive interventions during mechanical ventilation course

		**Day 1**	**Day 3**	**Day 5**	**Day 7**	**Day 10**	**Day 14**	**Day 21**
**Oxygenation status**								
PaO_2_, mmHg	**S**	77.0 [66.4–89.0]	85.0 [76.5–106.5]	82.0 [73.1–103.8]	81.2 * [76.7–99.1]	85.5 * [77.3–91.0]	92.5 * [83.2–106.5]	78.5 [70.9–105.6]
	**NS**	75.0 [61.0–91.8]	81.0 [64.2–103.0]	78.0 [60.6–97.3]	77.0 [65.0–83.8]	72.4 [61.7–79.5]	69.0 [55.3–78.0]	80.8 [76.9–87.3]
FiO_2_, unit	**S**	0.60 * [0.50–0.75]	0.50 * [0.48–0.60]	0.60 * [0.50–0.60]	0.50 * [0.41–0.55]	0.40 * [0.35–0.50]	0.40 * [0.31–0.49]	0.40 * [0.30–0.40]
	**NS**	0.75 [0.60–0.90]	0.70 [0.60–0.80]	0.70 [0.60–0.90]	0.65 [0.58–0.80]	0.80 [0.60–1.00]	0.85 [0.60–1.00]	0.70 [0.70–0.74]
PaO_2_/FiO_2_, mmHg	**S**	136.6 * [106.7–160.8]	170.6 * [124.2–210.0]	150.0 * [124.7–213.0]	161.7 * [141.6–245.2]	221.0 * [170.5–246.3]	252.7 * [169.8–316.7]	240.0 * [159.2–409.2]
	**NS**	105.5 [76.2–141.7]	125.0 [83.1–153.7]	112.0 [80.3–161.3]	108.9 [85.6–148.3]	92.2 [67.5–150.0]	79.0 [56.5–128.6]	116.2 [109.9–131.7]
«Optimum» PEEP, cmH_2_O	**S**	12.0 [10.0–12.0]	11.0 [10.0–14.0]	11.0 [10.0–14.0]	10.0 [10.0–12.0]	9.0 * [7.0–11.5]	8.5 [5.0–11.0]	7.0 [5.3–8.0]
	**NS**	11.0 [10.0–13.0]	12.0 [10.0–13.0]	10.0 [9.0–12.0]	10.0 [8.0–12.0]	10.0 [8.3–11.8]	10.0 [8.0–14.5]	9.5 [8.3–11.5]
**Ventilation status**								
Tidal volume, ml/kg IBW	**S**	8.2 [7.3–8.8]	8.1 [7.0–8.8]	8.2 [7.0–8.9]	8.0 [7.2–9.6]	8.1 [7.1–10.6]	7.6 [6.3–8.8]	7.9 [7.4–10.8]
	**NS**	7.7 [7.1–8.2]	7.7 [7.2–8.2]	7.9 [7.1–8.4]	7.9 [7.1–8.8]	7.9 [7.3–8.7]	8.2 [7.1–9.0]	7.8 [6.7–9.1]
Total RR, min^−1^	**S**	20 [18-22]	20 [18-22]	20 [18-22] *	21 [17-23]	20 [17-22] *	23 [20-25]	22 [15-23]
	**NS**	21 [18-24]	22 [18-24]	23 [20-26]	22 [18-25]	23 [19-25]	23 [20-28]	23 [22-29]
Minute ventilation, l/min	**S**	10.0 [8.5–12.0]	10.0 [8.6–11.3]	9.9 * [8.1–11.5]	10.6 [8.2–13.7]	10.6 [9.1–12.0]	11.1 [8.0–12.8]	12.1 [8.5–15.7]
	**NS**	11.0 [9.0–13.2]	10.7 [9.0–13.0]	11.7 [10.5–14.0]	11.5 [9.6–13.2]	12.2 [9.5–14.8]	12.6 [10.0–13.6]	10.7 [9.1–12.0]
PaCO_2_, mmHg	**S**	47.0 [40.0–61.4]	52.1 [39.3–61.6]	42.0 [38.0–55.2]	40.0 [34.6–45.6]	39.5 * [35.5–44.6]	40.0 [36.4–43.8]	43.6 [37.4–48.0]
	**NS**	47.9 [40.3–56.5]	49.5 [42.3–58.1]	46.4 [40.7–54.2]	45.0 [40.5–55.5]	47.0 [39.5–58.5]	48.0 [37.4–58.0]	45.8 [37.6–73.8]
Ventilatory ratio	**S**	1.9 [1.6–2.6]	1.9 [1.7–2.6]	2.0 [1.5–2.2]	1.9 [1.5–2.1]	1.8 [1.5–2.1] *	1.7 [1.6–2.1] *	2.2 [1.3–2.4]
	**NS**	2.2 [1.7–2.7]	2.2 [1.7–2.7]	2.2 [1.8–2.7]	2.1 [1.7–2.8]	2.5 [1.9–3.2]	2.5 [1.9–3.2]	2.7 [1.9–3.2]
VDalv/VT, %	**S**	0.39 [0.17–0.46]	0.38 [0.28–0.50]	0.33 [0.24–0.41]	0.24 * [0.19–0.31]	0.26 * [0.20–0.33]	0.30 [0.25–0.39]	0.30 [0.21–0.44]
	**NS**	0.36 [0.27–0.46]	0.38 [0.30–0.48]	0.37 [0.24–0.45]	0.37 [0.25–0.45]	0.40 [0.29–0.51]	0.29 [0.22–0.41]	0.31 [0.15–0.52]
**Respiratory mechanics**								
C_RS_, ml/cmH_2_O	**S**	38.4 [30.6–45.6]	37.0 [28.7–38.3]	39.8 [31.0–49.0]	42.2 * [29.4–49.0]	38.8 [28.2–45.3]	32.3 [25.3–46.3]	31.0 [25.0–43.0]
	**NS**	34.5 [25.0–42.3]	36.1 [26.2–42.3]	32.5 [25.0–41.5]	32.0 [24.5–41.8]	30.0 [26.0–37.3]	28.0 [20.7–43.5]	28.3 [15.0–39.0]
Plateau pressure, cmH_2_O	**S**	25.0 [23.5–27.5]	25.0 [24.0–30.0]	24.0 * [22.5–27.5]	23.0 * [22.0–25.0]	23.0 * [20.3–25.5]	22.5 * [19.5–27.3]	19.0 [15.0–19.0]
	**NS**	27.0 [24.0–31.0]	27.0 [24.0–30.0]	28.0 [24.0–31.5]	27.0 [24.0–31.0]	26.5 [24.0–30.8]	29.0 [24.5–36.5]	28.0 [22.3–34.5]
Driving pressure, cmH_2_O	**S**	13.5 [11.0–15.0]	14.0 [12.5–15.0]	13.0 * [10.5–15.0]	12.5 * [10.3–14.8]	13.5 * [11.0–15.8]	16.0 [11.5–17.5]	16.5 [13.0–22.3]
	**NS**	15.0 [12.0–20.0]	14.5 [12.0–19.0]	16.0 [13.0–20.0]	17.0 [13.5–20.5]	17.5 [15.0–20.0]	17.0 [13.0–27.5]	18.0 [11.8–25.8]
Resistance, mbar/l/s	**S**	10.0 [7.5–13.0]	10.0 [8.0–12.0]	10.5 [8.3–12.0]	8.5 [6.3–10.0]	9.5 [7.3–11.0]	10.5 [8.0–12.3]	11.0 [7.8–12.8]
	**NS**	9.0 [6.0–12.0]	9.0 [7.0–12.0]	8.0 [7.0–11.0]	8.0 [7.0–10.0]	8.5 [7.3–12.0]	10.0 [7.5–12.5]	8.0 [6.3–12.0]
**Adjunctive respiratory interventions**								
Neuromuscular blockade, n (%)	**S**	13 (76.5)	9 (52.9) *	6 (35.3) *	3(18.8) *	1 (6.3) *	1 (6.7) *	1 (14.3)
	**NS**	87 (87.9)	83 (84.7)	59 (80.8)	35 (71.4)	28 (87.5)	18 (85.7)	1 (25.0)
Prone position, n (%)	**S**	7 (41.2)	6 (35.3)	5 (29.4)	4 (25.0)	0 (0)	0 (0) *	0 (0)
	**NS**	49 (49.5)	35 (35.4)	22 (30.1)	8 (16.3)	3 (9.4)	5 (23.8)	1 (25.0)
Lateral position, n (%)	**S**	6 (35.3)	4 (23.5)	1 (5.9)	0 (0)	1 (6.3)	0 (0)	0 (0)
	**NS**	19 (19.2)	20 (20.2)	14 (19.2)	14 (28.6)	8 (25.0)	5 (23.8)	0 (0)
**Assessment of ventilator-associated respiratory infection**								
Modified CPIS, points	**S**	1 [1,2]	1 [1,2] *	2 [2] *	2 [2,3] *	5 [5]	5 [4,5]	5 [4,5]
	**NS**	1 [1,2]	2 [1,2]	2 [2,3]	3 [3,4]	5 [5,6]	5 [5,6]	6 [5,6]
**Organ support**								
ECMO, n (%)	**All**	0	0	0	0	0	0	0
RRT, n (%)	**S**	1 (5.9)	1 (5.9)	0	0	1 (6.3)	0 *	0
	**NS**	2 (2.0)	3 (3.1)	4 (5.5)	7 (14.3)	5 (16.1)	5 (23.8)	0
NE, n (%)	**S**	0	0 *	1 (5.9)*	1 (6.3)*	2(12.5)*	1 (7.1) *	0
	**NS**	19 (19.2)	27 (27.8)	32 (43.8)	21 (42.9)	18 (56.3)	14 (66.7)	2 (50.0)

Data presented as medians [interquartile range] or n (%) where appropriate. Differences between groups Mann–Whitney U-test, Chi-square or Fisher exact test where appropriate. Abbreviations:*S* Survivors; *NS* Non-Survivors; *PaO*_*2*_ arterial oxygen partial pressure; *FiO*_*2*_ fraction of inspiratory oxygen; *PEEP* positive end-expiratory pressure; *IBW* ideal body weight; *RR* respiratory rate; *PaCO*_*2*_ arterial carbon dioxide partial pressure; *VDalv/VT*; alveolar dead space to tidal volume ratio; *C*_*RS*_ compliance of the respiratory system; *CPIS* Clinical Pulmonary Infection Score; *ECMO* extracorporeal membrane oxygenation; *RRT* renal replacement therapy; *NE* norepinephrine

^*^p-value < 0.05, comparison between survivors and non-survivors

## Primary outcomes

**Fig. 5:**
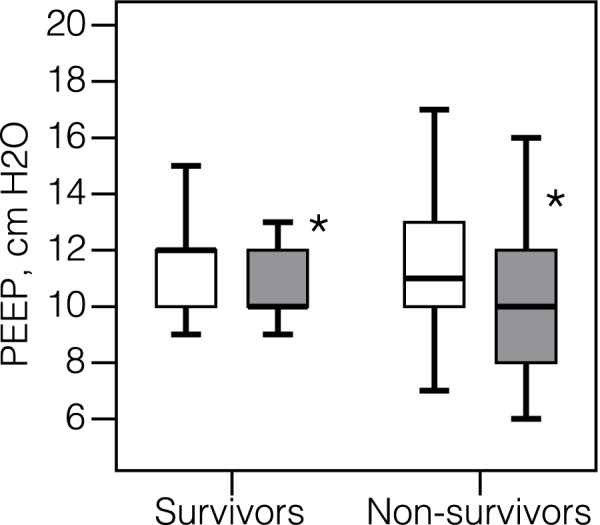
The «optimum» positive end-expiratory pressure (PEEP) levels on Day 1 and Day 7 in survivors and non-survivors. Data on Day 1(white) and Day 7 (grey) is presented as medians and 95% confidence intervals.* *p*-value < 0.05, comparison between Day 1 and Day 7 in subgroups of survivors and non-survivors

«Optimum» PEEP levels on Days 1 and 7 were 11.0 (10.0–12.8) cmH_2_O and 10.0 (9.0–12.0) cmH_2_O, respectively, with no differences between survivors and non-survivors (*p* = 0.705 and *p* = 0.835, respectively) (Fig. 5). The recruitment maneuver was effective in 29.3% of patients on Day 1, and in 9.2% on Day 7.

### Secondary outcomes

«Optimum» PEEP levels on Days 1, 3, 5, 7, 10, 14 and 21 are presented in Table 2. The «optimum» PEEP levels and tidal volumes were not different between survivors and non-survivors and within groups throughout the study.

Table 2 displays respiratory parameters, ventilatory settings and adjunctive interventions on days 1, 3, 5, 7, 10, 14 and 21 in all patients, survivors and non-survivors. The first day of mechanical ventilation in our study was associated with PaO_2_/FiO_2_ levels corresponding to moderate-to-severe ARDS (moderate ARDS 51.7%, severe ARDS 41.4%), high alveolar dead space with hypercapnia and high ventilatory ratio with a relatively preserved respiratory system compliance (Fig. 4, Table 2). Data on Day 1 was not significantly different between survivors and non-survivors except for the PaO_2_/FiO_2_ ratio that was lower in non-survivors (105.5 (76.2–141.7) mmHg vs 136.6 (106.7–160.8), *p* = 0.002).

Differences in PaO_2_/FiO_2_ ratio were significant between survivors and non-survivors at all study points. In survivors, PaO_2_/FiO_2_ ratio rose on Day 3 (*p *= 0.008) and then between Days 7 and 10 (*p* = 0.046). In non-survivors, PaO_2_/FiO_2_ ratio rose on Day 3 only (*p* = 0.013), decreased on Day 5 (*p* = 0.009), and later was stable until Day 10 and again decreased on Day 14 (*p* = 0.016 as compared to Day 10; *p* = 0.002—to Day 1).

Alveolar dead space was increased nearly two-fold in all patients, differences between survivors and non-survivors were significant on Days 7 and 10. Alveolar dead space decreased on Day 10 in survivors (*p* = 0.046 as compared to Day 1) and didn’t change in non-survivors. Accordingly, the ventilatory ratio was increased about two-fold in all patients and reached a statistical significance between survivors and non-survivors on Days 10 and 14 (*p* = 0.006 and *p* = 0.009, respectively) but failed to reach significance within subgroups in dynamics.

Patents who survived 28 days demonstrated a continuous increase of PaO_2_/FiO_2_ with a stable ventilatory ratio in survivors (*n* = 4), and a stable PaO_2_/FiO_2_ ratio with an increase of the ventilatory ratio in non-survivors after Day 10 (*n* = 4); survivors showed a relatively small decrease in static compliance after Day 7 with decrease in the «PEEP-dependency», while non-survivors showed a drop in static compliance with marked overdistension at «intermediate» PEEP levels (Table E1).

ICU and in-hospital mortality was 84.6% (*n* = 99). Non-survivors had a higher prevalence of cardiovascular and renal failure (Table 2). In survivors, the duration of mechanical ventilation was 15 [14–28] days and in non-survivors 7 [4–12] days. We didn’t find significant differences in days of noninvasive ventilation before intubation between survivors and non-survivors (3.0 (1.5–5.5) vs 3.0 (2.0–6.0) days, respectively, *p* = 0.831). We didn’t find any significant differences between survivors and non-survivors in the «non-respiratory» characteristics at baseline. The most valuable difference between groups during the observation period concerns catecholamine support—we can see a much higher need in norepinephrine administration in non-survivors from day 3 to day 14.

### ROC-analysis

ROC-analysis revealed that gas exchange parameters on Days 7 and 10 can be used as a prognostic point for mortality prediction: PaO_2_/FiO_2_ < 145 mmHg on Day 7 (Se 73.5%, Sp 75%, AUROC 0.82 (0.72–0.93), *p* < 0.0001) (Figure E1); VDalv/VT on Day 10 > 0.30 (Se 72%, Sp 75%, AUROC 0.80 (0.67–0.93), *p* = 0.001); Ventilatory ratio on Day 10 > 2.07 (Se 69%, Sp 75%, AUROC 0.75 (0.61–0.89), *p* = 0.006) (Figure E2). ROC-analysis for the static compliance measured at the preset PEEP and tidal volume and during the PEEP and volume trials showed non-significant results at all time points.

Laboratory values are presented in Table E2.

## Discussion

The results of our study can be summarized as follows: 1. All patients on Day 1 of invasive mechanical ventilation had PaO_2_/FiO_2_ levels corresponding to moderate-to-severe ARDS, nearly two-fold alveolar dead space and a slightly decreased respiratory system compliance at the tidal volume level; 2. On Day 1, we detected significant differences between survivors and non-survivors only in PaO_2_/FiO_2_; after Day 7, survivors had increased PaO_2_/FiO_2_ and decreased alveolar dead space irrespective of C_RS_ that remained stable after Day 7; 3. The potential for lung recruitment and response to the PEEP increase in COVID-19 was low, and it further decreased over time; 4. PEEP levels more than 10 cmH_2_O after Day 7 led to the lung overdistension in most patients; 4. Even a modest volume increase resulted in the lung overdistension that tended to increase over time.

After the publication on COVID-19-related L- and H-phenotypes [1], such phenotypes were identified in primary non-COVID ARDS [2]. In our study, we were unable to distinguish these phenotypes in mechanically ventilated patients but observed a slightly decreased compliance in all patients which is consistent with previous COVID reports [3–5] and the lung overdistension at intermediate tidal volumes.

According to Gattinoni L et al., L-phenotype lungs are not recruitable; later, after the development of ARDS (H-phenotype), dependent lung zones collapse and become susceptible to the PEEP rising [1]. In our patients, we observed a completely different picture, low recruitability at the beginning with completely non-recruitable lungs after one week of mechanical ventilation. Few data is available on the respiratory mechanics and response to PEEP in COVID-19 patients with acute respiratory failure [1, 3, 9, 10, 14–17]. Our results contradict previous data from Beloncle FM et al., where the majority of intubated patients were highly recruitable [10]. Although we used a different approach to assessing recruitment opportunities, this difference can be due to the fact that our study mainly included patients after NIV failure, while in the study by Beloncle et al. mechanical ventilation was the only treatment option. Of note, the high recruitability may be caused by the airway closure, rather than by lung recruitability per se [2, 11]. Haudeborg AF et al. observed that about 30% of COVID patients had recruitable lung [3], but 40% of these patients had airway closure [3, 11] as a marker of compression atelectasis, not alveolar collapse.

Observational studies comparing COVID with primary non-COVID ARDS found that COVID-ARDS was very close to ARDS due to bacterial and another virus pneumonia: slightly decreased compliance and driving pressure around 10 cmH_2_O with the disproportionately low PaO_2_/FiO_2_ [2]. Near-normal compliance in COVID-ARDS is in line with «baby lung» concept because «healthy» lung zones (in which the tidal volume is distributed (ventral parts predominantly)) have normal compliance [12]. This concept explains why the prone position can be effective in primary ARDS.

We hypothesized that SARS-CoV-2- pneumonia is a primary non-recruitable lung injury that results in a gradual decrease in the total vital lung capacity. Definitely, it’s very difficult to define lung overdistension based on the simple airway pressure measurement and usually requires extended respiratory monitoring (transpulmonary pressure and lung volumes measurements, quasi-static pressure–volume loop, etc.) to evaluate overdistension (stress and strain). The baby lung concept [13] showed us that lung restriction cannot be revealed if the tidal volume is less than functional residual capacity, so lung restriction and overdistension can be seen only during an increase of the tidal volume or PEEP (as a surrogate of the upper inflection point on a quasi-static pressure–volume loop). In these circumstances, lung compliance during tidal breath looks normal or slightly decreased, but a slight increase of PEEP or tidal volume reveals overdistension. We used a simplified bedside approach for assessment of lung overdistension using «the PEEP trial» and «the volume trial» that allow us to see a rapid increase in driving pressure (i.e. decrease in respiratory compliance) corresponding to lung overdistension. And we defined lung overdistension as an increase in driving pressure in these trials. So, our patients had lung overdistension after Day 5 in an «intermediate» PEEP and slightly increased tidal volumes despite plateau pressures less than 30 cmH_2_O in most measurements during tidal breath that corresponds to severe lung restriction and low lung recruitability. Also, we defined overdistension as an increase in driving pressure exceeding 15 cm H_2_O based on the study by Amato MB et al. [30] who found that value was associated with increased mortality in the analysis of randomized studies.

So, COVID-19 patients are at the high risk of ventilation-induced lung injury at intermediate PEEP or tidal volume levels like in other primary ARDS where PEEP generates injurious transpulmonary pressures and has a higher transmission to the pleura causing an increase in alveolar dead space [14]. Our data on PEEP levels is consistent with previous reports in similar respiratory mechanics, gas exchange at inclusion, and NIV usage before intubation which showed that higher PEEP levels (as in «higher» PEEP/FiO_2_ table) cause lung overdistension [6, 14, 15]. Studies on electro impedance tomography that looked for balance between lung recruitment and overdistension, revealed similar results: “optimum” PEEP levels around 12 cmH_2_O, lung overdistension at higher PEEP levels and the need in a personalized PEEP titration [16–18]. We agree with Sella N et al. [16] that PEEP/FiO_2_ tables should not be used in COVID-19-associated ARDS. Comparing primary ARDS of non-COVID and COVID origins Brault C et al., Grieco DL et al. and Chiumello D et al. found no major differences between them in the respiratory mechanics and gas exchange, high heterogeneity and the need for a personalized ventilation strategy [18, 20, 21], but they didn’t perform a «strain test» that can reveal the great volume differences in the rest of the «baby lung»***.*** Our “volume trial” found volutrauma at “intermediate” lung volumes that is rarely seen in secondary ARDS. Similar results were obtained in primary ARDS with the upper inflection point on the pressure–volume curve at tidal volumes around 600 ml [22].

Ventilation-perfusion mismatch and high alveolar dead space are of special interest in COVID-19 [23, 24]. We found a discrepancy between respiratory compliance and gas exchange in dynamics as survivors showed an increase in oxygenation and a decrease in the ventilatory ratio with a stable or even decreased respiratory compliance that can be partly explained by the restoration of lung perfusion and a ventilation-perfusion mismatch in survivors. Patel BV et al. found no correlations between computer tomography findings, PaO_2_/FiO_2_ or ventilatory ratio [24].

Recent studies have shown significantly lower mortality with early ECMO [25–27]. We suggest that COVID-19 patients without improvement in gas exchange and with signs of lung overdistension on «common» ventilatory settings on Day 7 should be considered for ECMO. Having very high mortality after NIV failure in these mechanically ventilated patients we can speculate that NIV failure in these patients can be one of the indications for ECMO. Maybe we should count NIV and invasive ventilation days together when we talk about the duration of mechanical ventilation before ECMO. The optimum time for switching from NIV to ECMO needs to be determined.

At a first glance, our study had higher mortality than others [4, 6]. We used invasive mechanical ventilation as the last step in the respiratory support chain after NIV failure in the conditions of ECMO shortage, not as the primary treatment option after low flow oxygen therapy had failed. Too many questions arose when we used intubation as the only option in primary ARDS based only on the PaO_2_/FiO_2_ ratio in patients without extrapulmonary organ failure and signs of impaired lung mechanics such as excessive respiratory muscle load on NIV or high-flow oxygen [28, 29].

Our study had several limitations. First of all, because it’s observational design. Second, it covered only patients in whom noninvasive ventilation failed and/or organ dysfunction, i.e. the most severe patients. Third, we didn’t measure additional physiologic parameters such as transpulmonary pressure, end-expiratory lung volume etc. Unfortunately, patients can die because of multiple organ failure due to resource shortage during a pandemic (ECMO, renal replacement therapy).

## Conclusions

Lung overdistension in COVID-19-associated acute respiratory distress syndrome occurs at «intermediate» positive end-expiratory pressures and tidal volume levels; it reflects severe lung restriction and a very high risk of volutrauma. In survivors gas exchange improvements occurred after Day 7 of mechanical ventilation and mismatched the respiratory compliance which didn’t increase but even decreased. Further research is warranted in order to decrease volutrauma and improve survival in COVID-19-associated ARDS.

## Supplementary Information

**Additional file 1.** Driving pressures (cmH_2_O) at «optimum» PEEP and the set tidal volume, during «PEEP trial» and «volume trial» during mechanical ventilation course

**Additional file 2.** Laboratory values during mechanical ventilation course

**Additional file 3.** ROC curve for mortality prediction for arterial oxygen partial pressure to the fraction of inspiratory oxygen ratio (PaO_2_/FiO_2_) on Day 7

**Additional file 4.** ROC curves for mortality prediction for the ventilation status parameters on Day 10

## Data Availability

The datasets used and/or analysed during the current study are available from the corresponding author on reasonable request.

## Abbreviations

ARDS: Acute respiratory distress syndrome
C_RS_: Static respiratory system compliance
DP: Driving pressure
FiO_2_: Inspiratory fraction of oxygen
ICU: Intensive care unit
PaCO_2_: Arterial carbon dioxide tension
PaO_2_: Partial pressure of oxygen in arterial blood
PBW: Predicted body weight
PEEP: Positive end-expiratory pressure
PetCO_2_: End-expiratory carbon dioxide tension
Pplat: Plateau pressure
PVI: Plethysmography variability index
RASS: Richmond Agitation-Sedation Score
RM: Recruitment maneuver
SpO_2_: Peripheral oxygen saturation
VDalv/VT: Alveolar dead space
VR: Ventilatory ratio
VT: Tidal volume
