# Supplementary material for: Potential for the lung recruitment and the risk of lung overdistension during 21 days of mechanical ventilation in patients with COVID-19 after noninvasive ventilation failure: the COVID-VENT observational trial
Source: BMC Anesthesiol. 2022 Mar 4;22:59. doi: 10.1186/s12871-022-01600-0 (PMC8894841; doi:10.1186/s12871-022-01600-0)
Supplement: Supplementary file 3 — Additional file 3. ROC curve for mortality prediction for arterial oxygen partial pressure to the fraction of inspiratory oxygen ratio (PaO2/FiO2) on Day 7 [file 12871_2022_1600_MOESM3_ESM.docx]

**Table E1. Driving pressures (cmH_2_O) at «optimum» PEEP and the set tidal volume, during «PEEP trial» and «volume trial» during mechanical ventilation course**

|  |  | **PEEP**  **8 cm H_2_O** | **PEEP**  **10 cm H_2_O** | **PEEP**  **12 cm H_2_O** | **PEEP**  **14 cm H_2_O** | **«Optimum» PEEP and VT** | **VT +100 ml** | **VT +200 ml** |
| --- | --- | --- | --- | --- | --- | --- | --- | --- |
| **Day 1** | **S** | 15.0 [12.0-16.5] | 14.0 [11.5-15.0] | 13.0 [12.0-16.5] | 15.0 [12.5-19.0] | 13.0 [11.5-15.0] | 17.0 [15.0-19.0] | 23.0 [20.0-28.0] |
|  | **NS** | 17.0 [14.0-20.0] | 16.0 [13.0-20.0] | 16.0 [13.0-21.0] | 18.0 [14.0-24.0] | 15.0 [12.0-20.0] | 20.0 [16.0-27.0] | 27.0 [21.0-37.0] |
| **Day 3** | **S** | 15.0 [12.0-16.5] | 14.0 [12.0-15.5] | 15.0 [12.0-17.0] | 16.0 [14.0-19.0] | 14.0 [12.5-15.0] | 18.0 [16.5-19.0] | 24.0 [20.5-25.5] * |
|  | **NS** | 15.0 [13.0-20.0] | 15.0 [12.0-19.0] | 16.0 [13.0-21.0] | 17.0 [14.0-21.0] | 14.5 [12.0-19.0] | 20.0 [16.0-25.0] | 26.5 [22.0-35.0] |
| **Day 5** | **S** | 12.5 [12.0-14.8] * | 12.5 [12.0-13.8] * | 13.0 [12.0-15.0] * | 15.5 [13.3-17.0] * | 13.0 [10.5-15.0] * | 17.0 [14.3-19.0] * | 24.0 [20.0-27.8] * |
|  | **NS** | 16.0 [13.0-20.0] | 17.0 [13.0-20.5] | 17.0 [13.0-22.0] | 19.0 [15.0-24.0] | 16.0 [13.0-20.0] | 21.0 [16.3-26.0] | 30.5 [23.0-37.8] |
| **Day 7** | **S** | 12.5 [10.5-14.0] * | 13.0 [10.3-13.5] * | 14.0 [10.5-17.3] * | 16.5 [13.3-18.5] * | 12.5 [10.3-14.8] * | 17.0 [14.3-18.8] * | 22.5 [20.0-28.3] * |
|  | **NS** | 16.0 [13.0-19.5] | 16.0 [13.0-21.0] | 17.0 [14.5-22.5] | 19.0 [15.5-23.5] | 17.0 [13.5-20.5] | 22.0 [18.0-29.0] | 30.0 [26.0-41.5] |
| **Day 10** | **S** | 13.5 [11.0-16.5] * | 13.5 [11.0-16.0] * | 15.0 [12.3-18.0] * | 16.5 [14.0-19.8] * | 13.5 [11.0-15.8] * | 17.0 [15.0-21.8] * | 23.0 [20.0-30.8] * |
|  | **NS** | 17.0 [15.0-18.8] | 17.0 [15.0-20.0] | 19.0 [17.0-23.0] | 20.0 [17.3-24.8] | 17.5 [15.0-20.0] | 22.0 [19.3-29.3] | 33.0 [26.0-38.5] |
| **Day 14** | **S** | 15.5 [11.5-18.5] | 16.5 [11.5-21.0] | 17.5 [12.5-23.8] | 19.0 [13.3-27.8] * | 16.0 [11.5-17.5] | 19.0 [14.8-24.3] | 25.5 [19.8-34.5] |
|  | **NS** | 18.0 [13.5-23.0] | 18.0 [12.5-25.0] | 23.0 [15.0-31.0] | 23.0 [16.5-32.5] | 17.0 [13.0-27.5] | 27.0 [18.0-36.0] | 36.0 [25.0-54.5] |
| **Day 21** | **S** | 17.0 [12.0-24.0] | 26.0 [12.0-26.0] | 24.0 [13.0-24.0] | 16.0 [15.0-16.0] | 16.5 [13.0-22.3] | 18.0 [16.0-18.0] | 24.0 [20.0-24.0] |
|  | **NS** | 18.5 [12.5-25.8] | 19.0 [11.8-29.3] | 28.5 [17.3-36.0] | 23.5 [11.5-41.5] | 18.0 [11.8-25.8] | 26.5 [15.5-48.8] | 37.0 [21.8-74.0] |
| **Day 28** | **S** | 19.0 [17.0-24.0] | 20.0 [17.0-20.0] | 20.5 [19.0-20.5] | 21.0 [20.0-21.0] | 19.0 [17.0-19.0] | 27.0 [24.0-27.0] | 37.0 [20.0-37.0] |
|  | **NS** | 38.0 [22.0-38.0] | 37.0 [24.0-37.0] | 42.5 [27.0-42.5] | 48.0 [31.0-48.0] | 30.0 [22.0-30.0] | 45.0 [36.0-45.0] | 68.5 [52.0-68.5] |

Data presented as medians [interquartile range]. Differences between groups by Mann-Whitney U-test.

**Abbreviations:** S: Survivors; NS: Non-Survivors; PEEP: positive end-expiratory pressure; VT: tidal volume.

* p-value < 0.05, comparison between survivors and non-survivors.
