# Supplementary material for: Potential for the lung recruitment and the risk of lung overdistension during 21 days of mechanical ventilation in patients with COVID-19 after noninvasive ventilation failure: the COVID-VENT observational trial
Source: BMC Anesthesiol. 2022 Mar 4;22:59. doi: 10.1186/s12871-022-01600-0 (PMC8894841; doi:10.1186/s12871-022-01600-0)
Supplement: Supplementary file 4 — Additional file 4. ROC curves for mortality prediction for the ventilation status parameters on Day 10 [file 12871_2022_1600_MOESM4_ESM.docx]

**Table E2. Laboratory values during mechanical ventilation course**

|  |  | **Day 1** | **Day 3** | **Day 5** | **Day 7** | **Day 10** | **Day 14** | **Day 21** |
| --- | --- | --- | --- | --- | --- | --- | --- | --- |
| **Hematology** | | | | | | | | |
| Hemoglobin, g/l | **S** | 116 [107-130] | 111 [101-119] | 110 [95-118] | 97 [86-110] | 98 [84-110] | 99 [83-113] | 90 [90-114] |
|  | **NS** | 123 [113-135] | 116[106-127] | 109 [98-123] | 106 [95-116] | 98 [87-114] | 93 [81-108] | 84 [68-89] |
| White blood cells, 10^9^/l | **S** | 9.6 [7.8-15.8] | 9.9 [7.8-12.3] | 8.8 [6.6-12.8] | 11.7 [7.8-14.9] | 9.9 [8.5-12.6] | 11.3 [7.5-12.7] | 10.1 [8.6-6.9] |
|  | **NS** | 11.5 [8.4-15.2] | 11.6 [9.3-15.4] | 11.1 [8.6-15.9] | 11.8 [8.8-16.1] | 11.5 [9.2-16.9] | 12.1 [9.5-17.2] | 14.6 [5.2-17.1] |
| Lymphocytes, 10^9^/l | **S** | 0.5 [0.5-0.8] | 0.6 [0.5-1.0] | 0.8 [0.5-0.9] | 0.9 [0.6-1.2] | 1.2 [0.8-1.3] | 1.0 [0.8-1.4] | 1.2 [1.1-1.5] |
|  | **NS** | 0.7 [0.4-0.9] | 0.8 [0.4-1.1] | 0.8 [0.6-1.3] | 0.7 [0.4-1.4] | 0.7 [0.6-1.1] | 0.7 [0.4-1.1] | 0.7 [0.4-1.4] |
| Platelets, 10^9^/l | **S** | 281 [177-412] | 363 * [172-442] | 317 * [254-457] | 325 * [171-412] | 310 § [201-388] | 236 [206-295] | 226 [188-396] |
|  | **NS** | 239 [167-288] | 202 [128-279] | 172 [114-269] | 181 [144-261] | 170 [106-230] | 184 [127-320] | 179 [81-242] |
| **Electrolites** | | | | | | | | |
| Sodium, mmol/l | **S** | 140 [136-145] | 142 [139-145] | 145 [140-148] | 140 * [138-142] | 141* [135-143] | 139 * [136-143] | 145 [147-155] |
|  | **NS** | 142 [137-145] | 145 [140-149] | 146 [140-151] | 147 [140-153] | 147 [139-158] | 145 [143-162] | 164 [148-171] |
| Potassium, mmol/l | **S** | 3.8 [3.7-4.4] | 4.0 [3.7-4.5] | 3.7 [3.3-5.0] | 3.7 * [3.5-4.0] | 4.0 *  [3.5-4.2] | 3.8 [3.6-4.2] | 3.8 [3.4-4.1] |
|  | **NS** | 4.2 [3.8-4.7] | 4.5 [3.8-5.2] | 4.4 [3.9-4.9] | 4.3 [3.7-5.2] | 4.4 [3.7-5.5] | 4.2 [3.7-5.6] | 3.5 [2.8-3.9] |
| **Biochemistry** | | | | | | | | |
| Albumin, g/l | **S** | 33.0 * [31.0-34.5] | 31.5 * [30.0-32.8] | 29.0 * [27.0-30.0] | 27.5 [23.0-30.3] | 28.0 [22.6-32.0] | 25.5 [22.7-31.5] | 28.0 [25.5-32.8] |
|  | **NS** | 29.0 [26.0-33.0] | 27.0 [25.0-30.0] | 25.9 [24.0-28.0] | 25.0 [23.0-28.0] | 24.0 [22.0-26.0] | 24.0 [21.5-27.0] | 29.0 [24.0-31.2] |
| LDH, U/l | **S** | 860 [716-1138] | 846 [638-1112] | 837 [695-975] | 661 [489-1013] | 683 * [585-820] | 606 [452-887] | 763 [560-865] |
|  | **NS** | 1026 [843-1429] | 1002 [746-1411] | 843 [655-1135] | 817 [713-1007] | 919  [712-320] | 1126 [703-1984] | 1031 [891-1134] |
| ALT, U/l | **S** | 43 [30-61] | 50 [27-93] | 46 [28-58] | 38 [24-62] | 38 [26-66] | 76 [29-118] | 35 [26-56] |
|  | **NS** | 37 [28-67] | 47 [30-78] | 37 [27-68] | 40 [26-69] | 37 [23-67] | 34 [24-76] | 61 [19-94] |
| AST, U/l | **S** | 37 [22-74] | 39 [25-56] | 32 [26-41] | 37 [23-63] | 45 [35-61] | 42 [29-80] | 28 [13-67] |
|  | **NS** | 48 [30-75] | 44 [31-83] | 45 [32-84] | 48 [36-81] | 53 [33-80] | 76 [31-139] | 62 [22-144] |
| Creatinine, µmol/l | **S** | 89 [66-114] | 95 [68-128] * | 102 [53-164] | 99 [68-163] | 74 * [62-99] | 77 [66-189] | 88 [71-272] |
|  | **NS** | 99 [76-139] | 133 [91-234] | 132 [95-220] | 141 [88-317] | 139 [91-293] | 127 [75-378] | 101 [56-292] |
| BUN, mmol/l | **S** | 8.2 [5.0-10.6] | 11.3 * [7.0-15.5] | 11.0 [8.2-17.9] | 9.7 * [8.0-18.0] | 9.2 § [5.6-14.8] | 8.0 [5.1-22.8] | 6.0 [4.3-23.0] |
|  | **NS** | 10.0 [8.0-15.0] | 17.0 [10.0-24.3] | 19.0 [10.3-30.5] | 19.5 [9.4-46.3] | 22.0 [11.2-37.3] | 14.0 [8.5-28.1] | 15.5 [10.8-36.0] |
| Total bilirubin, µmol/l | **S** | 10.6 [7.7-18.1] | 9.3 [6.4-13.5] | 6.4 [5.3-10.7] | 7. 5 [5.6-11.8] | 7.6 [6.6-11.5] | 6.8 [5.6-11.5] | 6.0 [5.2-16.2] |
|  | **NS** | 11.0 [7.0-15.0] | 10.9 [7.0-15.0] | 8.0 [6.7-15.5] | 10.0 [6.1-13.4] | 9.0 [7.0-12.0] | 8.6 [5.9-13.5] | 10.0 [8.0-13.2] |
| CRP, mg/l | **S** | 163 [118-234] | 173 [105-219] | 102 [53-164] | 137 [80-185] | 66 * [45-134] | 125 [61-158] | 94 * [30-130] |
|  | **NS** | 157 [98-258] | 126 [73-220] | 126 [49-240] | 187 [63-232] | 173 [113-249] | 164 [68-307] | 174 [122-273] |
| Ferritine, mg/l | **S** | 529 [347-597] | 498 [373-564] | 466 [247-529] | 227 [82-444] | 281 [100-520[ | 455 [348-564] | 370 [190-432] |
|  | **NS** | 483 [306-524] | 442 [228-515] | 333 [228-507] | 290 [219-520] | 500 [284-523] | 295 [148-525] | 497 [273-513] |
| **Coagulation** | | | | | | | | |
| INR, % | **S** | 1.2 [1.1-1.4] | 1.2 [1.2-1.3] | 1.3 [1.2-1.5] | 1.4 [1.2-1.5] | 1.3 [1.2-1.5] | 1.4 [1.2-2.0] | 1.4 [1.2-1.9] |
|  | **NS** | 1.2 [1.1-1.4] | 1.3 [1.1-1.4] | 1.3 [1.1-1.5] | 1.3 [1.2-1.6] | 1.3 [1.2-1.8] | 1.2 [1.0-2.4] | 1.8 [1.2-2.1] |
| aPTT, s | **S** | 39.0 [32.5-43.8] | 42.0 [30.5-44.0] | 38.0 [32.0-48.0] | 46.5 [35.5-64.5] | 38.0 [34.0-47.5] | 42.0 [35.0-52.5] | 48.0 [40.4-78.0] |
|  | **NS** | 39.0 [35.0-47.0] | 39.5 [35.0-48.6] | 42.0 [37.0-56.0] | 49.0 [38.5-63.2] | 44.5 [37.1-60.8] | 46.0 [36.0-94.0] | 48.4 [37.3-56.0] |
| D-dimers, mcg/l | **S** | 3.3 [2.0-6.0] | 1.8 [1.0-9.4] | 1.6 [1.3-3.7] | 1.8 [0.9-4.3] | 2.2 [1.3-5.1] | 2.4 [1.5-5.3] | 3.3 [1.1-5.0] |
|  | **NS** | 3.3 [1.6-7.3] | 3.7 [1.5-6.6] | 2.3 [1.3-3.9] | 2.0 [1.1-4.3] | 2.0 [1.6-3.6] | 2.7 [1.5-5.8] | 2.1 [1.8-3.7] |
| Fibrinogen, g/l | **S** | 6.9 [5.3-10.6] | 6.2 [5.4-9.8] | 6.4 [5.5-8.9] | 7.2 [4.7-9.8] | 6.1 [4.5-8.9] | 4.7 [4.1-7.3] | 5.3 [3.6-8.3] |
|  | **NS** | 7.6 [5.7-9.4] | 6.5 [4.8-8.7] | 6.5 [4.4-9.6] | 7.2 [4.6-9.9] | 7.5 [5.6-9.0] | 7.7 [4.6-11.3] | 6.9 [5.8-12.9] |

Data presented as medians [interquartile range] or n (%) where appropriate. Differences between groups Mann-Whitney U-test.

**Abbreviations:** S - Survivors; NS- Non-Survivors; LDH- lactate dehydrogenase; AST - aspartate aminotransferase; ALT - alanine aminotransferase; BUN - blood urea nitrogen; CRP- C-reactive protein; INR - international normalized unit; aPTT - activated partial thromboplastin time.

* p-value < 0.05, comparison between survivors and non-survivors.
